# Artificial Pancreas and Closed-Loop Insulin Delivery: From Early Concepts to AI-Driven Diabetes Automation

**DOI:** 10.3390/bios16090507

**Published:** 2026-09-10

**Authors:** Reem Emad Al-Dhaleai, Mustafa Tariq Khan, Zaid Chilmeran, Abdulrahman Husain AlSadeq, Alexandra E. Butler

**Affiliations:** 1 School of Medicine, Royal College of Surgeons in Ireland-Medical University of Bahrain, Busaiteen P.O. Box 15503, Bahrain; zaidchilmeran@outlook.com (Z.C.); 22203638@rcsi-mub.com (A.H.A.); 2 Research Department, Royal College of Surgeons in Ireland-Medical University of Bahrain, Busaiteen P.O. Box 15503, Bahrain; abutler@rcsi.com

**Keywords:** artificial pancreas, closed-loop insulin delivery, automated insulin delivery, continuous glucose monitoring, model predictive control, diabetes technology, machine learning

## Abstract

Closed-loop insulin delivery, or the artificial pancreas, has evolved from an ambitious engineering concept into one of the most consequential advances in diabetes technology. By integrating continuous glucose monitoring, insulin pumps, and control algorithms into a single feedback system, these platforms aim to shift diabetes care from repeated manual correction toward more anticipatory and adaptive glucose regulation. The field has progressed from early proof-of-concept systems to contemporary hybrid closed-loop platforms, reflecting a deeper shift in diabetes management itself: from treating glucose excursions after they occur to trying to blunt them in real time. Its clinical relevance is greatest in type 1 diabetes, where the burden of self-management is high and the consequences of glycemic instability are immediate. This review introduces the major technologies and evidence shaping the field, with emphasis on the control strategies that drive system behavior, and asks which currently available closed-loop systems offer the best balance of glycemic benefit, usability, and translational readiness for routine diabetes care. Across randomized trials and real-world studies, automated insulin delivery has consistently improved time in range, reduced hypoglycemia, and enhanced patient experience, particularly in pediatric populations. At the same time, important limitations remain: sensor lag, the physiologic constraints of subcutaneous insulin, device complexity, cost, and unequal access limit full autonomy and widespread adoption. Looking ahead, the next phase of progress will likely depend on more adaptive artificial intelligence, improved meal detection, multimodal wearable data, and multi-hormone systems that move the field closer to truly physiologic glucose control.

## 1. Introduction

Closed-loop insulin delivery has rapidly evolved from experimental prototypes to commercially available hybrid systems, yet the evidence base remains difficult to interpret because studies differ in device architecture, algorithmic control, degree of automation, and outcome reporting. As a result, clinicians and patients are often left with a practical question that is not answered by individual trials alone: among the systems currently available, which ones provide the best overall balance of glycaemic benefit, usability, and feasibility for routine implementation? This question matters because the success of automated insulin delivery depends not only on improvements in time in range (TIR) or HbA1c, but also on whether the technology can be used consistently in everyday life, accepted by patients, and supported within real-world care pathways.

The current commercial landscape includes platforms that vary in how much insulin adjustment is automated, how much user input is required, and how well they have been studied across different age groups and clinical settings. Some systems prioritize tighter glycemic control, whereas others emphasize simplicity, safety, or interoperability with existing devices. These differences make direct comparison essential. Accordingly, this review introduces the available literature to identify which closed-loop systems currently offer the most favorable combination of glycemic efficacy, user burden, and translational readiness, and to highlight the remaining barriers that must be addressed before fully autonomous insulin delivery can become routine clinical care.

## 2. Methods

**Figure 1:**
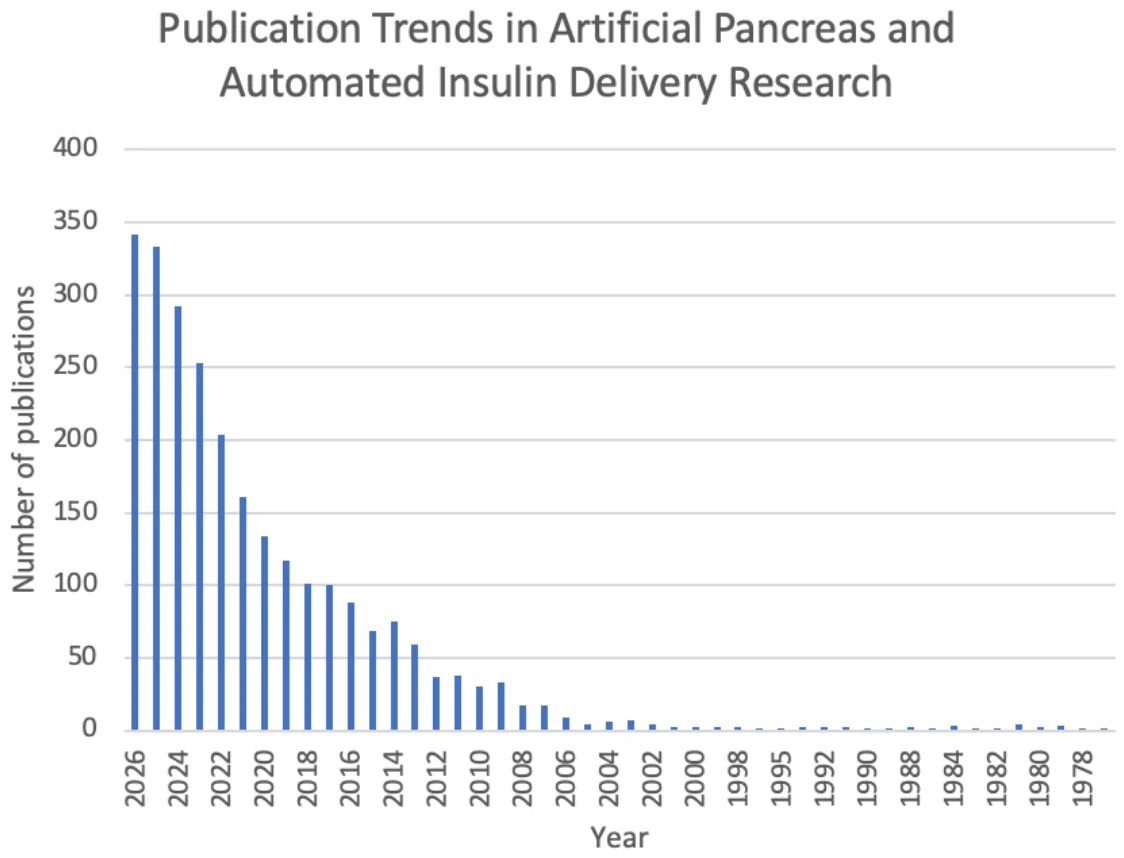
Publication trends in artificial pancreas and automated insulin delivery research, 1978–2026. Data were obtained from PubMed using the search strategy described in Section 2 and analyzed in Microsoft Excel; 2026 represents publications indexed up to August 2026.

A literature search was performed using PubMed to identify relevant publications on the historical development of closed-loop insulin delivery, control algorithms, commercial AID systems, clinical outcomes, implementation challenges, and emerging artificial intelligence applications. Search terms included combinations of artificial pancreas, closed-loop insulin delivery, automated insulin delivery, hybrid closed-loop, continuous glucose monitoring, control algorithms, MiniMed 780G, Control-IQ, Omnipod 5, CamAPS FX, and machine learning. A literature search was performed using PubMed to identify relevant publications on the historical development of closed-loop insulin delivery, control algorithms, commercial AID systems, clinical outcomes, implementation challenges, and emerging artificial intelligence applications. The following search strategy was used: (“artificial pancreas” OR “closed-loop insulin delivery” OR “automated insulin delivery” OR “hybrid closed-loop” OR “closed loop” OR “automated insulin delivery system” OR “AID”) AND (diabetes OR “type 1 diabetes” OR T1D) AND (“continuous glucose monitoring” OR CGM OR “insulin pump” OR “control algorithm” OR “model predictive control” OR PID OR “machine learning” OR “reinforcement learning” OR “meal detection” OR exercise OR “physical activity” OR wearable OR multimodal OR “multi-hormone”). The review focused primarily on recent randomized controlled trials, systematic reviews, meta-analyses, consensus statements, clinical guidelines, and real-world studies, while foundational studies were included to provide historical and technological context. A publication trend analysis was also performed using the records retrieved from PubMed using the search strategy described above (Figure 1). The search results were exported from PubMed in CSV format, and the number of publications by year was extracted and analyzed in Microsoft Excel.

## 3. Evolution and Working Principles of Artificial Pancreas Systems

### 3.1. Historical Development

Closed-loop insulin delivery, also known as the artificial pancreas, is one of the most important developments in modern diabetes technology because it transforms insulin therapy from a largely manual process into a dynamic feedback system. In its simplest form, the technology combines continuous glucose monitoring (CGM), an insulin pump, and a control algorithm so that insulin delivery can be adjusted automatically in response to sensed glucose levels [1]. What makes this approach so consequential is not only its technical design, but the conceptual shift it represents: instead of asking patients to continuously correct glucose level excursions after they occur, closed-loop systems aim to anticipate and blunt dysglycemia in real time. The concept of the artificial pancreas is described as a long-standing goal of diabetes care that only became clinically feasible once glucose sensing, insulin delivery, and algorithmic control matured enough to function together in everyday use [2,3]. Although the field has advanced from early proof-of-concept systems to commercially available hybrid closed-loop platforms, the literature remains fragmented across devices, algorithms, and real-world implementation settings.

**Figure 2:**
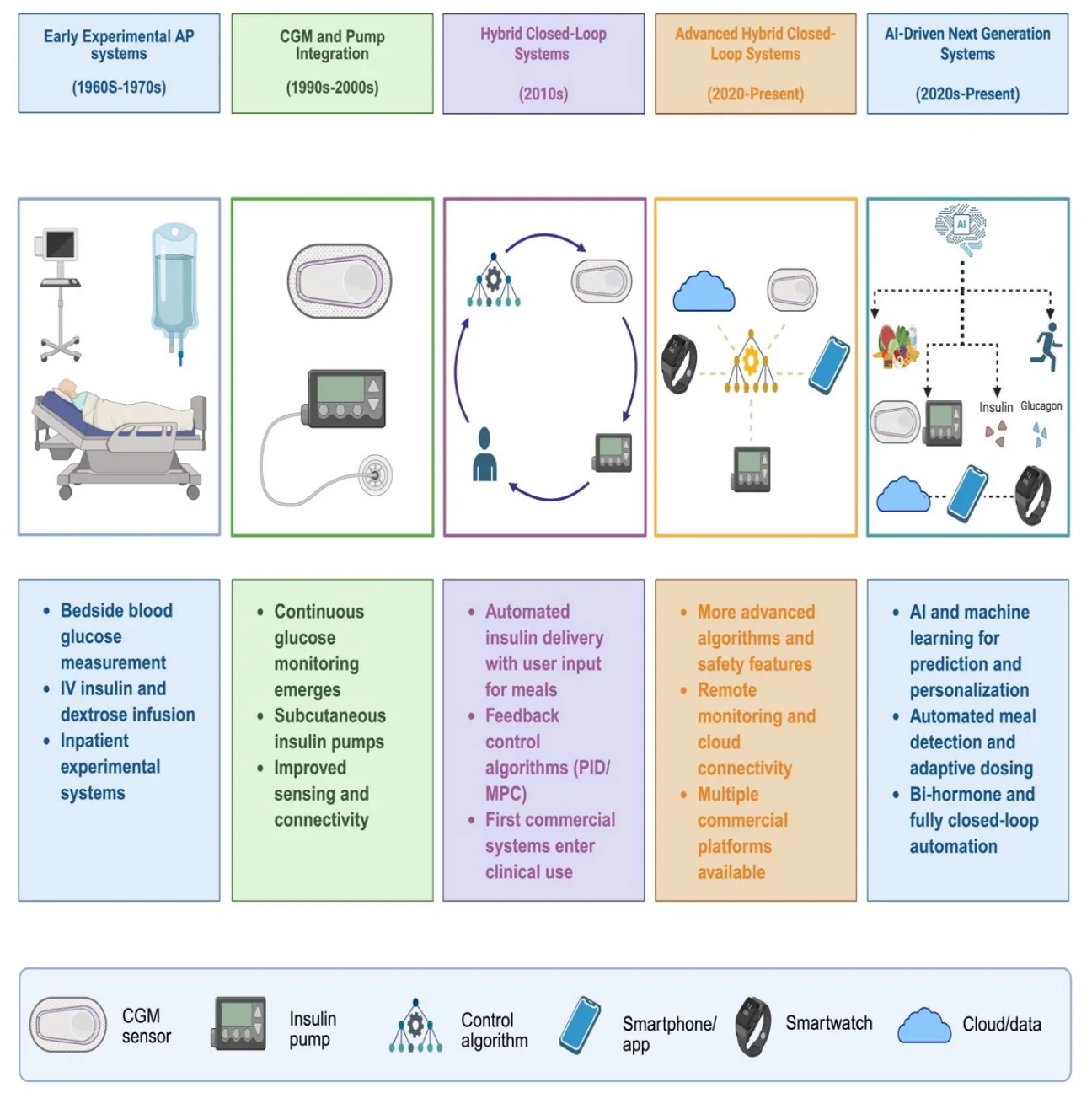
Evolution of artificial pancreas technology from early experimental closed-loop systems to AI-driven diabetes automation. Created in https://BioRender.com.

The development of the field has been gradual but transformative (Figure 2). Early closed-loop systems, first explored in the 1960s and 1970s, relied on bedside glucose measurements and intravenous insulin–dextrose infusions to maintain near-normal glycemia. These proof-of-concept systems demonstrated that glucose-responsive insulin delivery was possible, but they were too bulky, too slow, and too impractical for routine outpatient care. Over time, the field moved toward wearable devices, subcutaneous insulin pumps, and interstitial CGM, allowing closed-loop therapy to shift from laboratory experimentation to real-world diabetes management. This evolution is well summarized in many reviews that trace the pathway from early prototypes to today’s commercially available hybrid closed-loop systems [2,3]. Early artificial pancreas systems, first explored in the mid-20th century, were, as previously mentioned, proof-of-concept devices designed to demonstrate that glucose-responsive insulin delivery was possible. These systems were scientifically important, but they remained far from practical outpatient use because they depended on cumbersome hardware, delayed glucose measurements, and delivery methods that were not suited to daily life. What has made the field truly transformative is the convergence of more accurate CGM, portable insulin pumps, and increasingly sophisticated control algorithms, allowing closed-loop therapy to evolve from a laboratory aspiration into a clinically deployable technology. Recent reviews describe this shift as the key moment when closed-loop systems stopped being simply experimental and began functioning as real diabetes management platforms [3,4,5,6,7].

This evolution matters because diabetes, particularly T1D, is not a disease that can be managed effectively by occasional correction alone. Patients must continuously balance basal insulin requirements, meal-related glucose excursions, physical activity, illness, stress, and the ever-present risk of hypoglycemia. In that sense, closed-loop insulin delivery represents more than convenience; it represents a new model of insulin therapy in which glucose regulation becomes increasingly anticipatory rather than purely reactive. Many reports emphasize that AID reduces treatment burden while improving glycemic outcomes, and they now frame these systems as an established part of contemporary diabetes technology rather than a futuristic concept. This is especially important in T1D, where the burden of self-management is greatest and the margin for error is narrowest [3,8,9].

Seen from this perspective, the artificial pancreas is not simply a device innovation, but a conceptual shift in the way insulin therapy is delivered. Instead of relying on repeated human correction, it moves care toward prediction, adaptation, and partial autonomy. That is why the field has attracted so much attention: it sits at the intersection of endocrinology, biomedical engineering, and computational control, and it has already begun to reshape what “good diabetes management” looks like in practice. The literature increasingly treats AID as a bridge toward more physiologic insulin replacement and, potentially, toward even more autonomous systems as sensing, modeling, and algorithmic control continue to improve.

The clinical importance of this technology is greatest in people who require intensive insulin therapy, especially those with type 1 diabetes (T1D). Diabetes self-management demands constant decisions about basal insulin, mealtime dosing, exercise, illness, and hypoglycemia prevention, which makes treatment both cognitively and physically burdensome. Automated insulin delivery (AID) reduces that burden while improving glycemic control, and that is why it is increasingly viewed not simply as another glucose-lowering tool, but as a major step toward more physiologic insulin replacement and more autonomous diabetes care. Contemporary consensus reports also note that regulatory approval and clinical adoption of AID systems have accelerated over the last decade, marking the transition of closed-loop therapy from a research ambition to an established part of diabetes practice [3,10].

### 3.2. Glucose Monitoring and Insulin Delivery

CGM provides the primary feedback signal for AID systems. CGM systems measure glucose concentrations in the interstitial fluid through a subcutaneous sensor and continuously transmit the resulting data to a receiver, smartphone, or insulin-delivery controller. This continuous stream allows the system to assess not only the current glucose concentration but also the direction and rate of glucose change, which are important for automated insulin adjustment [3,11]. However, because CGM measures interstitial rather than blood glucose, a physiological and measurement delay exists between changes in blood glucose and the sensor-derived signal. This delay becomes particularly relevant during rapid changes in glucose concentration, such as following meals or during physical activity, and can therefore affect the timing of automated insulin delivery [12].

Insulin pumps provide the therapeutic component of the closed-loop system by delivering rapid-acting insulin into the subcutaneous tissue. Conventional pump therapy allows programmed basal insulin delivery together with user-initiated bolus doses for meals and correction of hyperglycemia. In AID systems, the control algorithm uses CGM-derived glucose information to adjust insulin delivery automatically. Depending on the system, this may involve increasing or decreasing basal insulin delivery and, in advanced hybrid systems, delivering automated correction boluses, while users may still be required to announce meals and administer prandial insulin [3,11].

The fundamental closed-loop architecture therefore consists of three interconnected components: a glucose sensor that provides continuous feedback, a control algorithm that interprets current and predicted glucose trajectories, and an insulin-delivery system that modifies insulin administration in response [3,11]. The process can be represented as a continuous feedback loop in which CGM data are transmitted to the controller, the controller determines the appropriate insulin response, and the resulting change in glucose is subsequently detected by the sensor. The effectiveness of this loop depends on the performance of each component and on how well the system compensates for sensor and insulin-action delays.

A further limitation arises from the pharmacokinetic and pharmacodynamic properties of subcutaneous insulin. Rapid-acting insulin analogues used in AID have an onset of action of approximately 10–15 min and peak activity after approximately 1–2 h, meaning that insulin delivered in response to a rising glucose concentration cannot immediately reproduce the rapid physiological insulin response to a meal [12]. The combination of interstitial glucose sensing delay and delayed subcutaneous insulin action therefore represents a fundamental constraint on the speed and precision of current closed-loop systems and helps explain why contemporary systems remain predominantly hybrid rather than fully autonomous.

### 3.3. Current Closed-Loop Systems

Among current commercial platforms, MiniMed 780G, Tandem Control-IQ, Omnipod 5, and CamAPS FX are particularly useful for comparison because they represent different design choices, different degrees of automation, and different evidence profiles; a recent network meta-analysis of 28 randomized trials, all featuring people with T1D, found that MiniMed 780G achieved the highest TIR among the reviewed hybrid closed-loop systems, ahead of Control-IQ, while all systems reduced time below range and showed similar rates of severe hypoglycemia and diabetic ketoacidosis [13].

Although these platforms share the same basic closed-loop architecture, they are not functionally identical. Their differences lie in how aggressively they automate insulin delivery, how much user input they still require, and how they balance safety against glycemic tightness. That distinction matters because the term “hybrid closed-loop” can obscure meaningful differences in daily experience, especially when comparing systems that prioritize strong automation, tubeless design, or lower user burden [13,14,15].

## 4. Algorithmic Approaches

The control algorithm is the intellectual core of the artificial pancreas. Sensors and pumps provide the hardware, but the algorithm determines whether the system behaves like a cautious insulin calculator, a predictive controller, or an adaptive learning machine. This is why the evolution of artificial pancreas research has not been driven by one “best” algorithm, but by a continuing effort to solve a biologically difficult problem: glucose regulation is delayed, nonlinear, highly individualized, and shaped by unpredictable disturbances such as meals, exercise, illness, and stress. Reviews of closed-loop insulin delivery repeatedly emphasize that the central challenge is not simply delivering insulin automatically, but delivering it in a way that is safe, stable, and responsive to changing physiology [16,17,18].

PID control is one of the most established strategies in the field. Its appeal lies in its simplicity: it responds to the current glucose error, the accumulated past error, and the direction or speed of change, which makes it intuitive and relatively easy to implement. In a closed-loop setting, that simplicity is valuable because it offers transparency and robustness. However, PID also reveals the limitations of a purely reactive approach. It can respond only after glucose has already moved away from the target, which makes it less effective for rapid postprandial excursions or situations in which glucose changes faster than insulin can act. For that reason, PID remains an important conceptual foundation, but one that is often surpassed by more predictive methods when the goal is tighter, more anticipatory control [16,18,19].

MPC represents a more ambitious and arguably more elegant solution. Rather than waiting for glucose to drift out of range and then correcting it, MPC uses a mathematical model to forecast where glucose is likely to go and then selects the insulin action most likely to keep values near target over a future time horizon. This predictive architecture is one reason MPC has become so influential in artificial pancreas research. An in silico trial of T1D showed that MPC could outperform PID by better handling meal announcement information, delay compensation, and glucose oscillations, while many reviews describe it as one of the most practical and promising strategies for closed-loop insulin delivery. Its weakness is also its strength: because it depends on a model, performance can deteriorate when the model does not fit the individual’s true insulin sensitivity, meal behavior, or daily activity pattern. In other words, MPC brings the system closer to intelligent regulation, but only as long as the model remains close enough to the patient’s physiology [16,17,19]. This theoretical strength may be the reason for its translation into measurable clinical differences. In a randomized crossover study directly comparing the two approaches, namely MPC and PID, personalized MPC achieved a higher TIR than PID (74.4% vs. 63.7%, *p* = 0.020) and lower glucose concentrations throughout the whole trial (138 vs. 160 mg/dL, *p* = 0.020) and 5 h after an unannounced 65-g meal (181 vs. 220 mg/dL, *p* = 0.019); however, the time with glucose <70 mg/dL showed no significant difference [20].

Fuzzy logic and rule-based systems approach the same problem from a different angle. Instead of relying heavily on equations, they encode human reasoning into a set of interpretable rules, making them attractive where clinical intuition and personalization are important. This is especially useful in diabetes care, where physicians often tailor insulin decisions based on glucose goals, meal patterns, lifestyle, and hypoglycemia risk. Papers on fuzzy logic artificial pancreas systems show that these controllers can be individualized through physician tuning and personalization factors, which makes them conceptually appealing for bedside adaptation. At the same time, the literature also makes clear why fuzzy systems never became the sole dominant paradigm: they are harder to scale, tune, and generalize than model-based approaches, and their performance can depend heavily on how well the rules capture real-world variability. For that reason, fuzzy logic is best understood not as a replacement for model-based control, but as an important attempt to bring expert reasoning into automation [21,22,23,24].

The newest direction is adaptive and machine-learning-based control, which tries to move the artificial pancreas beyond fixed rules and toward systems that learn from data. Reinforcement learning (RL) is especially attractive because it is designed for sequential decision-making, making it conceptually well matched to insulin delivery, where every action affects future glucose trajectories. Reviews of RL in diabetes describe its potential to individualize insulin dosing and improve safety, while broader machine-learning reviews in glucose prediction emphasize that data-driven methods may improve forecasting of glycemic excursions and hypoglycemia risk. More recent experimental work has started to test these ideas in practice: a randomized crossover trial of a neural-net artificial pancreas introduced a new paradigm for AID, and other studies have used machine learning (ML) to model glucose dynamics and reduce hypoglycemia risk. In a pilot randomized crossover study, the implementation of a neural-network controller achieved similar glycemic outcomes to the established MPC algorithm, achieving a TIR of 86.1% versus 87.5% and a time below 70 mg/dL of 1.9% versus 1.8% while requiring one-sixth of the computation. These findings are suggestive of the fact that the near-term role of machine learning may be to enhance the efficiency and scalability of currently proven control-strategies rather than to replace model-based control [25]. Yet the promise of AI should not be overstated. These methods still face major barriers, including safety validation, transparency, robustness to unannounced meals and exercise, and the need for long-term clinical evidence rather than short feasibility studies. The field is therefore entering a new phase: not whether algorithms can learn, but how they can learn safely enough to become trustworthy clinical tools. Taken together, these algorithmic approaches show that the artificial pancreas is not a single technology but a spectrum of control philosophies, ranging from reactive regulation to predictive optimization and, increasingly, to adaptive learning systems that may eventually make insulin delivery genuinely intelligent [25,26,27,28,29].

## 5. Current Challenges and Emerging Directions in AI-Driven Automated Insulin Delivery

### 5.1. Current Challenges

Despite increasing interest in machine-learning and reinforcement-learning approaches, several important challenges continue to limit fully autonomous insulin delivery. A major issue is that in many systems, glucose prediction and insulin dosing remain largely dependent on information about the latest glucose concentrations, insulin delivery, and manually reported carbohydrate consumption. However, it is important to note that carbohydrates do not alone contribute to the metabolic effects of a meal, and fats and proteins can alter the magnitude and timing of the postprandial glycemic excursions and may result in delayed or prolonged increases in insulin requirements. Systematic reviews have clearly demonstrated that meals comprising varying levels of fat, protein, and glycemic index may require different insulin-delivery patterns, highlighting the pitfalls of a carbohydrate-focused approach, as high-fat and high-protein meals may produce delayed and prolonged postprandial glucose excursions that are not readily captured by carbohydrate quantity alone, while the optimal timing and magnitude of additional insulin requirements remain uncertain [30,31]. This creates an important challenge for automated insulin delivery, because an algorithm that does not account for these nutritional effects may respond too late or inadequately to the subsequent glucose excursion. Incorporating broader information about meal composition into future prediction and insulin-dosing models may therefore improve the ability of AID systems to anticipate postprandial responses and provide more individualized insulin delivery.

Current AI-based approaches have begun to address a related component of this problem by reducing the need for manual meal announcement; as an example, Mosquera-Lopez et al. developed a neural-network-based “robust artificial pancreas” (RAP) capable of detecting meals from CGM and insulin-delivery data and estimating the carbohydrate content required to recommend a meal bolus. For algorithm development, the authors used data from 199 virtual subjects simulated for 14 days using two validated type 1 diabetes simulators, including the FDA-approved UVA-Padova simulator. The model used 32 features derived from the preceding 2 h of CGM and insulin data, including average glucose, glucose rate of change, insulin availability and time of day. The meal-detection algorithm achieved a sensitivity of 83.3%, a false-discovery rate of 16.6%, and a mean detection time of 25.9 min. The approach was subsequently evaluated in a randomized, single-center crossover study involving 15 adults, of whom 13 completed the study, with type 1 diabetes, in which the RAP system was compared with hybrid model-predictive control following unannounced meals. RAP significantly reduced time above range by 10.8% (*p* = 0.04), while time in range increased by 9.1% but did not reach statistical significance (*p* = 0.09); there was no significant difference in time below range. These findings demonstrate the feasibility of using machine learning to reduce reliance on manual meal announcement. However, the use of simulated populations during algorithm development also highlights the need for careful real-world validation, as virtual cohorts cannot fully reproduce the physiological, behavioral and environmental variability encountered in people with diabetes. The remaining detection delay and reliance on carbohydrate estimation further illustrate the challenges that must be overcome before fully autonomous meal management can be achieved [32].

Recent reinforcement-learning has extended this concept by incorporating specific metabolic contexts into individualized insulin dosing. Jafar et al. developed a reinforcement-learning decision-support system for high-fat meals and postprandial aerobic exercise. In a single-arm 16-week proof-of-concept study involving 15 adults with type 1 diabetes, the system was associated with improved postprandial glucose exposure and reduced time spent below 3.9 mmol/L following high-fat meals and meals followed by exercise. However, the study was designed primarily to assess feasibility, and larger randomized trials are required before the approach can be considered established [33].

Physical activity represents a second major source of uncertainty. Exercise can cause substantial glucose fluctuations, and responses vary according to the type, intensity and timing of activity as well as insulin availability and individual physiological characteristics. A joint EASD/ISPAD position statement published in 2025 identified physical activity as a continuing challenge for current automated insulin-delivery systems and noted that users still commonly need to anticipate activity and make adjustments around planned exercise. Future algorithms may therefore benefit from incorporating activity-related information from accelerometry, heart rate and other wearable-derived signals into glucose prediction and insulin-delivery decisions [34].

The increasing complexity of AI models has also increased interest in explainable artificial intelligence (XAI), as complex models may provide limited insight into the factors underlying individual recommendations, which may pose challenges for clinical trust, auditing and safety. Recent diabetes research has begun to incorporate explainability directly into insulin-management models. He et al. developed an expert-guided XAI framework for insulin titration that used the Shapley Taylor Interaction Index to capture feature interactions and incorporated clinician feedback through a doctor-in-the-loop process. The framework produced explanations that were more closely aligned with expert reasoning and improved insulin-titration accuracy among junior clinicians, while both junior and senior clinicians reported increased confidence in the system [35]. Although this work was performed in insulin titration for type 2 diabetes rather than a closed-loop artificial-pancreas system, it still illustrates the emerging role of XAI in making increasingly complex diabetes algorithms more transparent and clinically interpretable.

### 5.2. Future Directions: Towards Fully Autonomous and Multimodal Automated Insulin Delivery

The next generation of AID is likely to focus on reducing the remaining need for manual intervention while improving the ability of algorithms to anticipate rather than simply respond to metabolic disturbances. Current hybrid closed-loop systems have demonstrated substantial clinical benefit, but meal announcement and carbohydrate estimation remain important components of routine use. Fully closed-loop insulin delivery therefore represents an important research objective. In a randomized crossover study of 35 adults with type 1 diabetes, Garcia-Tirado et al. compared hybrid closed-loop, fully closed-loop, and fully closed-loop control incorporating meal anticipation. Although meal anticipation did not significantly improve postprandial time in range compared with fully closed-loop control, the fully automated approaches achieved overall 24-h time in range above 70%, demonstrating the feasibility of reducing reliance on manual meal announcements while also highlighting the difficulty of predicting postprandial glucose excursions [36].

More recently, Pryor et al. evaluated AIDANET, a miniature neural-network-based controller designed to facilitate fully closed-loop insulin delivery, in a randomized crossover pilot study involving six adults with type 1 diabetes. The system achieved a time in range of 66.4% compared with 63.3% during usual care, with no serious adverse events reported; however, the small sample size and short follow-up mean that these findings should be regarded as preliminary feasibility evidence rather than evidence of established fully autonomous therapy [37].

The future objective is not simply automated meal detection, but meal-aware prediction that can translate the characteristics of a meal into an individualized, time-dependent insulin-delivery strategy. Real-world datasets may also contribute to this development. Lu et al. used more than 45,000 meal records from 82 individuals to develop personalized long short-term memory models for predicting mealtimes, illustrating how longitudinal patient data could potentially support individualized anticipatory control [38].

In parallel, dual- and multi-hormone closed-loop systems continue to evolve. Bihormonal artificial pancreas systems incorporating both insulin and glucagon have demonstrated improvements in glycemic outcomes while reducing the risk of hypoglycemia. In a recent one-year study conducted in the Netherlands, participants using a bihormonal system achieved approximately 80% time-in-range with very low rates of hypoglycemia [39]. Evidence from this study suggests that the addition of glucagon may provide further protection against hypoglycemia compared with insulin-only systems. However, wider adoption remains limited by factors such as increased system complexity and the need for stable glucagon formulations, including newer analogues such as dasiglucagon. Additional research is also exploring the incorporation of hormones such as amylin and glucagon-like peptide-1 (GLP-1) receptor agonists to more closely replicate physiological glucose regulation [40].

Another important area of development is AI-driven personalization of insulin therapy. Adaptive algorithms, including RL models, are being investigated for their ability to adjust insulin dosing based on individual responses to factors such as meal composition, physical activity, and daily behavioral patterns. Building on early proof-of-concept evidence, such approaches could enable AID systems to move beyond population-based settings towards more individualized treatment strategies [33]. Furthermore, future controllers may, therefore, use longitudinal information on meals, exercise and glycemic responses to refine insulin dosing dynamically. Such adaptation will nevertheless require explicit safety constraints, because an algorithm that modifies its own dosing policy must remain stable and safe when exposed to novel or poorly represented physiological conditions.

Integration of multimodal wearable data represents a related avenue for increasing contextual awareness. CGM and insulin-delivery data provide the core inputs for current AID, but information from accelerometers, heart-rate monitors, sleep trackers and other wearable sensors may provide additional context regarding factors that influence glucose variability. Fraser et al. reviewed 60 studies examining the integration of AI and wearable technology in diabetes and identified potential applications in personalized monitoring and intervention while also highlighting limitations related to data quality, demographic representation, external validation and interpretability [41]. Collectively, these studies illustrate the potential for wearable-derived behavioral and physiological signals to complement conventional glucose-based monitoring and support more proactive diabetes management [41,42].

A further unmet need is the development of truly multianalyte wearable sensor networks that extend beyond continuous glucose monitoring to capture ketones and metabolic or hormonal signals relevant to glucose regulation. Continuous ketone monitoring could provide an important safety layer for AID by identifying rising β-hydroxybutyrate concentrations before diabetic ketoacidosis becomes clinically apparent and enabling ketone information to be incorporated alongside glucose data into automated decision-making. Expert consensus has specifically proposed integration of continuous ketone monitoring with CGM and automated insulin-delivery systems, while recent reviews have highlighted the broader potential of multianalyte sensors to simultaneously measure glucose and other biomarkers such as ketones and insulin [43,44]. This approach is becoming increasingly tangible and is supported by recent human studies demonstrating wearable continuous β-hydroxybutyrate monitoring using a microneedle-based platform, although integration of ketone measurements directly into closed-loop insulin-delivery algorithms remains an important area for further study [45]. Beyond ketones, continuous monitoring of insulin and counter-regulatory hormones such as glucagon and cortisol could, in principle, provide direct information about insulin availability, endogenous counter-regulation and stress-related metabolic disturbances that are currently inferred indirectly from glucose and behavioral signals. Wearable insulin biosensors and continuous cortisol-sensing platforms remain largely investigational, however, and reliable long-term monitoring of these hormones presents substantial challenges in sensor specificity, calibration, biofluid sampling and physiological interpretation [46,47]. A future multimodal AID network could therefore combine glucose, ketones, insulin, glucagon, cortisol, physical activity and other contextual signals to improve individualized prediction and safety, potentially helping compensate for the delays inherent in subcutaneous insulin delivery. Such systems will require rigorous validation to determine which analytes provide clinically actionable information and whether the additional physiological information translates into tighter glycemic control without increasing system complexity or false interventions.

As AID systems become more autonomous and computationally complex, explainable AI is likely to become increasingly important. An algorithm that influences insulin delivery without direct user input must operate safely under uncertainty while remaining sufficiently transparent to permit clinical oversight and post hoc auditing. The earlier referenced He et al. study cannot be said to demonstrate that explainable AI has been established in artificial-pancreas systems, despite reporting improved alignment between model explanations and expert reasoning and improved insulin-titration accuracy among junior clinicians [35], because this study used hospitalized patients with type 2 diabetes and addressed insulin titration rather than closed-loop AID. Instead, it illustrates a broader direction in diabetes AI toward incorporating clinical knowledge and interpretability into increasingly complex models. For future AID, similar principles could support systems in which automated decisions are accompanied by interpretable information about the variables driving a recommendation, measures of uncertainty and predefined safety constraints.

Taken together, the next generation of artificial-pancreas systems is likely to be defined by the integration of several developments rather than by the emergence of a single superior algorithm. Fully closed-loop control could reduce dependence on manual meal announcements; meal-aware algorithms could incorporate nutritional and temporal characteristics of food; adaptive methods could personalize insulin delivery to changing individual responses; multimodal sensing could provide information about exercise and other contextual disturbances; and multi-hormone systems could provide additional physiological control when insulin alone is insufficient. The principal translational challenge will be to combine these capabilities without introducing unacceptable complexity, instability or safety risk. Future studies should therefore prioritize prospective evaluation in diverse real-world populations, independent external validation, clinically meaningful endpoints and transparent safety assessment so that increasing algorithmic autonomy is accompanied by evidence of reliability and clinical benefit. The goal of future AID should not simply be to automate more decisions, but to develop systems that can make increasingly informed decisions safely across the unpredictable conditions of everyday diabetes management.

## 6. Evidence from Clinical Trials

The strongest clinical evidence for AID is in T1D, where the technology has moved from experimental promise to reproducible clinical benefit. Across randomized trials, AID improves TIR, lowers glycemic risk, and reduces both hyperglycemia and hypoglycemia without a clear safety penalty. A 2025 systematic review and meta-analysis of 65 randomized trials involving 3623 participants found that AID increased TIR by 11.74% and improved glycemia risk index, with particularly favorable effects in people with longer diabetes duration and in dual-hormone systems. That matters because it shows the field is not just improving glucose numbers in the abstract; it is improving control in the very patients most exposed to glycemic instability [48].

**Figure 3:**
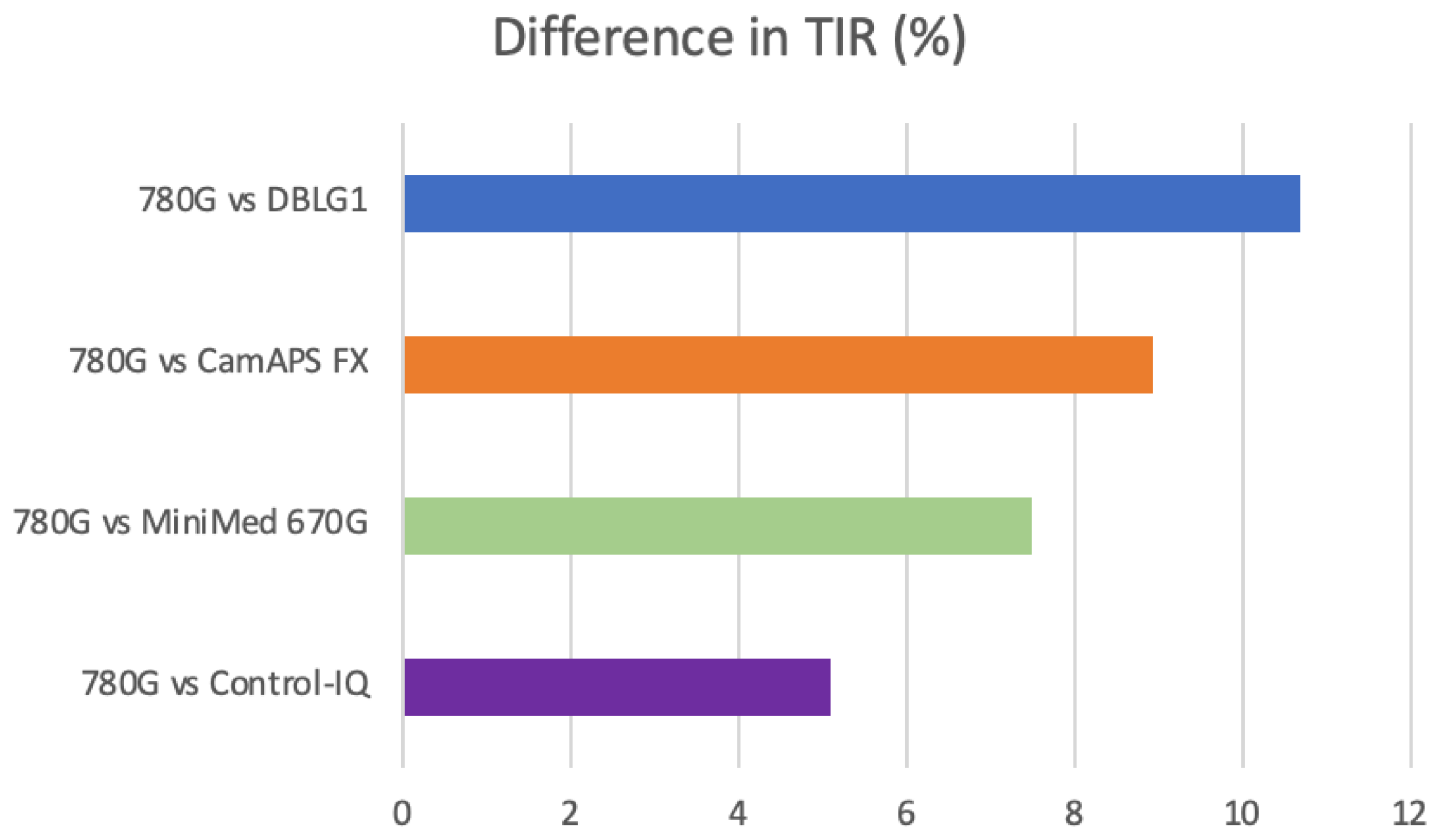
Differences in Time in Range Between MiniMed 780G and Other Hybrid Closed-Loop Systems.

**Figure 4:**
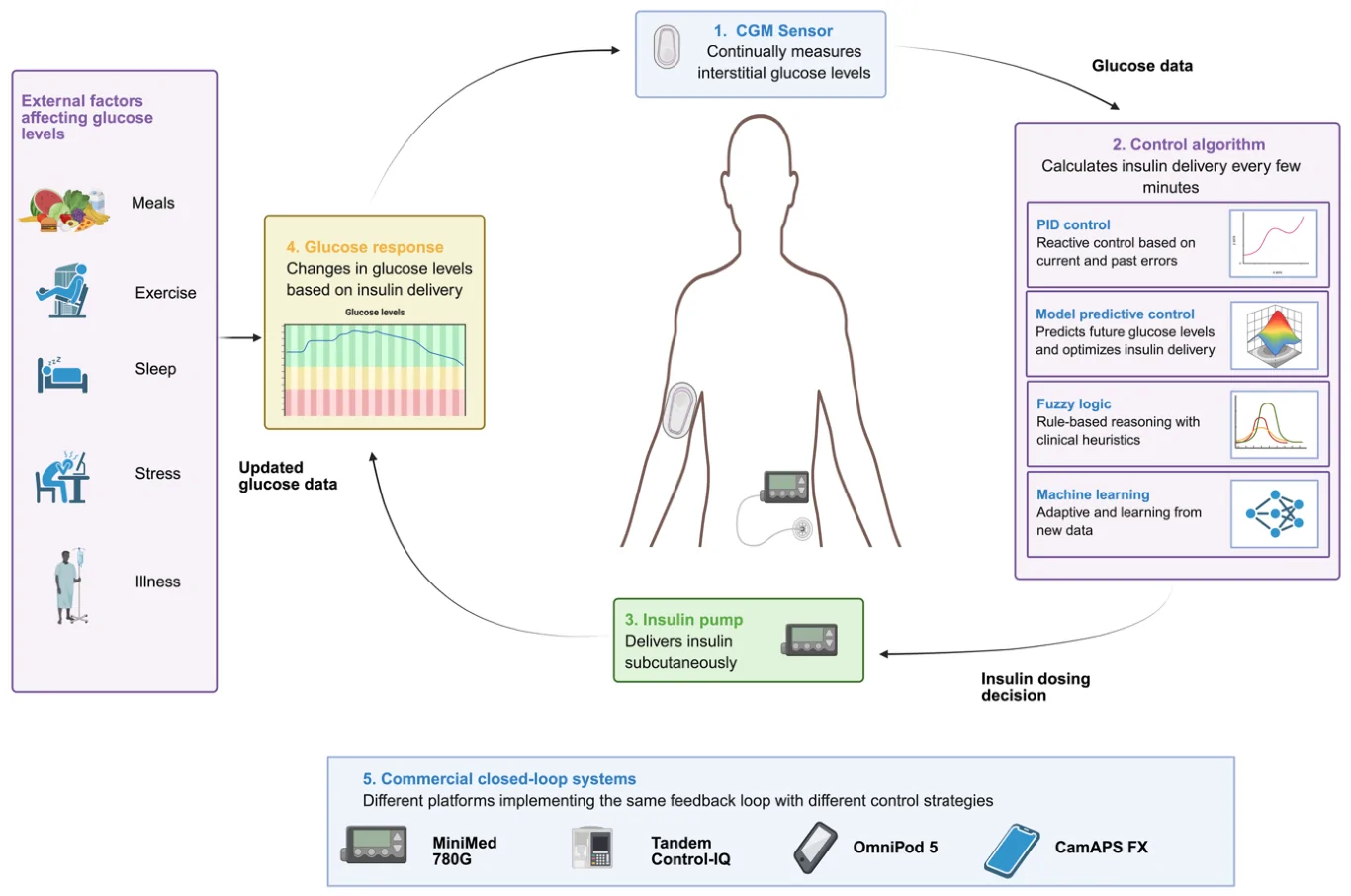
Architecture and workflow of contemporary closed-loop insulin delivery systems. Created in https://BioRender.com.

Hybrid-loop systems significantly increased the TIR compared with subcutaneous insulin therapy and no continuous glucose monitoring. MiniMed 780G achieved the highest TIR, ahead of Control-IQ, MiniMed 670G, CamAPS FX, and DBLG1; the mean differences in time in range versus subcutaneous insulin therapy without continuous glucose monitoring were 5.1% for Control-IQ, 7.48% for MiniMed 670G, 8.94% for CamAPS FX, and 10.69% for DBLG1. All hybrid closed-loop systems also decreased time below target range, with the largest reductions seen with DBLG1, MiniMed 670G, and MiniMed 780G. It is important to note that the risk of severe hypoglycemia and diabetic ketoacidosis was similar across systems and comparable to other insulin therapies, suggesting that current hybrid closed-loop systems share broadly similar safety profiles, but differ meaningfully in the degree of glycemic benefit they achieve [13]. MiniMed 780G appears especially strong in comparative analyses of time in range (Figure 3); Control-IQ has robust real-world durability data showing sustained improvement over one year; Omnipod 5 shows rapid early glycemic improvement in real-world use; and CamAPS FX stands out for pregnancy data, where randomized evidence has shown a meaningful increase in time in range compared with standard insulin therapy. Therefore, a more informative way to read the trial literature is by platform rather than by diagnosis alone (Figure 4, Table 1). Taken together, these studies suggest that the clinical question is no longer whether AID works, but which system performs best for which population and under which real-world conditions [13,14,49,50].

The pediatric evidence is especially strong (Table 2). In a 2025 systematic review and meta-analysis of randomized trials in youth aged 6 to 18 years, AID reduced glycated hemoglobin (HbA1c) by 0.41% and increased time in range by 11.5% while also reducing time spent in both hypoglycemia and hyperglycemia. Importantly, these gains came without an increase in adverse events, which strengthens the case that the benefit is not purchased at the cost of safety. What makes this especially persuasive is that the improvement was not limited to a single outcome: the systems consistently shifted the glucose profile toward more stable, less hazardous control [54].

Real-world pediatric studies make the same point in a more clinically vivid way. In the National Health Service (NHS) England hybrid closed-loop cohort of 251 children and young people with T1D, 12 months of use was associated with a 7 mmol/mol reduction in HbA1c, a 13.4% increase in time in range, and a 50% reduction in hypoglycemia frequency. These gains were accompanied by better sleep and less fear of hypoglycemia for both patients and caregivers, which matters because the value of diabetes technology is not confined to lab values; it also includes how much it changes the burden of living with the disease. A separate 1-year real-world multicenter study of 368 children and adolescents using MiniMed 780G found sustained improvement in HbA1c and time in range, with higher time in range linked to fewer SmartGuard exits, fewer automatic correction boluses, and longer time spent in automatic mode. That adds an important nuance: clinical benefit is strongest when the system is actually allowed to stay active and do its job [55,57].

Adult studies broaden the picture rather than merely repeating it (Table 3). In real-world use, hybrid closed-loop systems have been associated with sustained glycemic and patient-reported benefits in adults with T1D, suggesting that the technology can work beyond carefully selected trial populations. Evidence in type 2 diabetes is smaller but still important: a randomized trial in insulin-treated adults with type 2 diabetes found that closed-loop insulin delivery without mealtime boluses was effective and safe in the general ward, showing that the concept is not confined to T1D. Taken together, the trial literature now supports a strong conclusion: AID has crossed the threshold from promising innovation to clinically meaningful therapy while still leaving open questions about which system, which population, and which implementation model produces the best outcome [58,59,60].

## 7. Implementation, Usability, and Translational Barriers

### 7.1. Real-World Implementation and Access

The most interesting question is no longer whether AID works, but how well it fits the realities of ordinary life. Real-world performance depends on more than algorithm accuracy or pump design; it depends on whether the system can be woven into school schedules, work routines, sleep, exercise, illness, and the ordinary unpredictability of meals. That is why implementation studies matter: they reveal the distance between clinical efficacy and lived usability, and they show that the artificial pancreas is still a human–machine partnership rather than a fully autonomous solution [57].

Patient experience is one of the clearest strengths, but also one of the clearest reminders that these systems are not invisible. Qualitative and psychosocial data show benefits such as improved well-being, greater flexibility, and better collaboration with care teams, yet they also describe technical glitches, device maintenance, bulk and visibility of devices, frequent alarms, and the burden of handling large volumes of data. Those trade-offs matter because a system can be clinically effective and still feel demanding to use. In pregnancy-focused literature, these burdens are especially visible, but they reflect a broader truth about closed-loop therapy: the technology reduces workload in some domains while introducing new forms of attention and troubleshooting in others [64,65].

Those differences become more apparent when systems are used outside ideal settings. Even in strong real-world cohorts, benefit is linked to staying in automated mode, minimizing exits, and maintaining consistent sensor and pump use. In the 1-year pediatric MiniMed 780G study, better time in range was associated with fewer SmartGuard exits and longer time in automatic mode, which shows that performance depends on sustained interaction between the user and the system. Adult real-world studies likewise report durable benefits, but they also confirm that closed-loop therapy is not a “set and forget” intervention; it still requires training, troubleshooting, and continued engagement [57].

Acceptability also varies by platform. Omnipod 5 is especially useful to discuss here because real-world and psychosocial studies show not only improved glycemia but also better diabetes distress, sleep quality, system usability, and insulin-delivery satisfaction for children, adolescents, and caregivers. That makes it a strong example of a system whose value extends beyond glucose metrics into the lived experience of diabetes care [49,66,67]. In contrast, large real-world cohorts for Control-IQ and MiniMed 780G show sustained glycemic benefit over time, which suggests high practicality and continued use, even when the psychosocial literature is less prominent [14,57,68,69].

Access may be the most important barrier of all. Reviews of diabetes technology disparities in adolescents and young adults describe unequal uptake and persistent barriers to access and use, while clinical reviews of hybrid closed-loop systems explicitly discuss access and reimbursement as part of the current landscape. This is where the literature becomes especially important for a review paper: the field is no longer limited by what the devices can do in principle, but by who can realistically receive them, learn them, afford them, and keep them running over time. In that sense, future progress will depend not only on smarter algorithms, but also on improved implementation, greater equity in access, and more effective integration of diabetes technologies into health systems [70,71].

### 7.2. Technical and Physiological Limitations

Despite the substantial clinical benefits associated with artificial pancreas systems, technological limitations continue to constrain their performance and practicality in everyday use. Current closed-loop systems rely heavily on commercially available CGM, insulin pumps, and external computing devices, meaning that overall system effectiveness is dependent on the capabilities and reliability of each individual component. As a result, hardware malfunctions, connectivity issues, and communication failures can disrupt AID and require user intervention. Wireless communication between sensors, pumps, and controllers introduces additional vulnerabilities, particularly when signal interruptions or device pairing failures occur. Battery life also remains an important consideration, as continuous operation of multiple interconnected devices can increase power demands and reduce convenience for users. Furthermore, the hormonal therapies used within these systems are limited by currently available formulations [11].

Another important limitation of artificial pancreas systems is the accuracy and physiological delay associated with CGM technology, which serves as the foundation for AID. Unlike conventional blood glucose measurements, CGM devices measure glucose concentrations within the interstitial fluid, resulting in an inherent lag between sensor readings and actual blood glucose levels. This delay typically ranges from 3 to 12 min and becomes most significant during periods of rapid glycemic fluctuation, such as after meals, during exercise, or following insulin administration. As a result, closed-loop algorithms may respond to glucose values that do not accurately reflect the individual’s current metabolic state, potentially compromising glycemic control. Sensor performance may also be affected by calibration errors, delayed user calibration, signal-processing limitations, measurement noise, and temporary signal loss. Although advances in sensor technology have substantially improved accuracy, these challenges remain clinically relevant. Earlier hybrid closed-loop studies reported sensor failures and signal interruptions that affected system performance, highlighting the dependence of AID on reliable glucose monitoring [12].

Beyond technological and sensor-related constraints, the pharmacokinetic and pharmacodynamic properties of currently available insulin formulations represent a fundamental limitation of artificial pancreas systems. In individuals without diabetes, insulin is secreted directly into the portal circulation in a highly coordinated manner, allowing rapid responses to changes in blood glucose levels, particularly following meals. This physiological regulation cannot be fully replicated in closed-loop systems, as insulin is delivered subcutaneously and must first be absorbed into the circulation before exerting its effects. Although rapid-acting insulin analogues such as lispro, aspart, and glulisine have improved the speed of insulin delivery, their onset of action typically occurs after 10–15 min, with peak activity reached approximately 1–2 h later. Furthermore, attempts by automated algorithms to compensate for rising glucose levels may result in larger corrective insulin doses, increasing the risk of delayed postprandial hypoglycaemia once insulin action reaches its peak. As a result, the physiological limitations of subcutaneous insulin delivery continue to restrict the ability of artificial pancreas systems to achieve truly physiological glucose regulation [12].

Finally, despite the clinical benefits associated with artificial pancreas systems, their cost remains a significant barrier to widespread adoption. Although ongoing animal studies investigating artificial pancreas patches have reported projected costs of approximately $10 per device [72], the success and future commercial availability of these technologies remain uncertain. In contrast, currently established artificial pancreas and closed-loop insulin delivery systems typically cost between $3000 and $8000 [73], creating a substantial financial burden for patients and healthcare systems. Furthermore, the expense extends beyond the initial purchase of the device. Real-time CGM, which is essential for the operation of these systems, costs approximately $10 per day, amounting to around $14,000 over four years. Additional consumable components, including infusion sets and reservoirs, contribute a further estimated cost of $7000 over the same period. The cumulative financial demands of closed-loop therapy may lead insurers and healthcare providers to impose restrictions on patient eligibility and access [74].

## 8. Conclusions

Artificial pancreas systems have transformed diabetes management by integrating CGM, insulin delivery, and automated control into a unified therapeutic platform. The accumulated evidence now indicates that the central question is no longer whether automated insulin delivery is effective, but rather which systems provide the optimal balance of glycemic benefit, usability, and accessibility for different patient populations. The superiority of advanced hybrid closed-loop systems over conventional pump therapy is well established, with improvements in time in range typically approaching 20–25 percentage points in recent network meta-analyses, albeit with meaningful variation among platforms [75].

At the same time, the remaining barriers to fully autonomous glucose regulation are increasingly translational rather than conceptual. Limitations in sensor accuracy, delays in subcutaneous insulin action, device burden, cost, and inequitable access now represent the principal obstacles to broader implementation. Future progress will therefore depend not only on more sophisticated algorithms and multi-hormone approaches, but also on making these advances safe, interpretable, scalable, and widely available. In this sense, the evolution of the artificial pancreas reflects a broader shift in diabetes care: from demonstrating technological feasibility to ensuring that the benefits of automation can be delivered equitably and sustainably in routine clinical practice.

## Figures and Tables

**Table 1:** Comparison of Currently Available Hybrid Closed-Loop Platforms.

System	Algorithm	Insulin Delivery Characteristics	User Input Required?	Distinguishing Features
MiniMed 780G	Modified proportional–integral–derivative (PID) algorithm incorporating features of the MD-Logic artificial pancreas algorithm	Automatic correction boluses every 5 min; selectable glucose targets of 100, 110, or 120 mg/dL [51].	Meal announcement and carbohydrate estimation required	One of the most automated commercial systems with aggressive correction capability and strong real-world evidence
Tandem Control-IQ	Model Predictive Control (MPC)	Automated insulin adjustments and correction dosing based on predicted glucose trends [52].	Meal boluses required	Strong long-term real-world data and broad adoption across age groups
Omnipod 5	MPC-based adaptive algorithm located directly within the pod	Automated micro-bolus delivery every 5 min using a 60-min prediction horizon [53].	Meal boluses required	Only widely available tubeless AID platform; user-adjustable glucose target of 110–150 mg/dL
CamAPS FX	Adaptive Model Predictive Control algorithm	Continuously adjusts insulin delivery using adaptive predictions [52].	Meal announcement required	Particularly notable for evidence in pregnancy and highly individualized glucose control

**Table 2:** Pediatric clinical evidence comparison by device.

Study/Population	Device	Key Outcome
251 children and young people with T1D in the NHS England pilot	Hybrid closed loop (Tandem Control-IQ (78%), Minimed 780G (11%), and CamAPS FX (11%))	HbA1c fell by 7 mmol/mol, time in range rose by 13.4%, and hypoglycemia frequency fell by 50%; sleep and fear of hypoglycemia improved [55].
368 children and adolescents using MiniMed 780G	MiniMed 780G	HbA1c and time in range improved over 1 year, and better outcomes were linked to more time in automatic mode and fewer SmartGuard exits [49].
Youth real-world use of Control-IQ	Control-IQ	Glycemic control improved and system use remained high at 6 months [56].
First pediatric users/early real-world Omnipod 5 use	Omnipod 5	Favorable glycemic outcomes in pediatric users, with early real-world benefit [56].

**Table 3:** Adult clinical evidence comparison by device.

Study/Population	Device	Key Outcome
Pregnant women with T1D	CamAPS FX	Around a 10% increase in time in pregnancy range compared with standard insulin therapy [50].
Pregnant women with T1D using Control-IQ off label	Control-IQ	Lower odds of large-for-gestational age (LGA) infants [61].
Adults with insulin-treated type 2 diabetes	Omnipod 5	Extended use improved glycemic outcomes over 34 weeks, with a decrease in percentage of time ≥ 250 mg/dL from 27.4% ± 21.0% to 10.5% ± 8.8% [62].
Adults with T1D in real-world use	Control-IQ	One-year use was associated with improved glycemic management and better quality of life, with fewer school/work absences [63].

## Data Availability

No new data were created or analyzed in this study. Data sharing is not applicable to this article.
